# Predicting Functional Decline and Falls in Older Adults Participating in Gerofit

**DOI:** 10.1093/geroni/igaf122.2449

**Published:** 2025-12-31

**Authors:** Ben Friedman, Jamie Giffuni, Megan Kelly, Julie Rekant, Odessa Addison

**Affiliations:** University of Maryland, Baltimore County, Baltimore, Maryland, United States; Veterans Affairs, Baltimore, Maryland, United States; Veterans Affairs, Baltimore, Maryland, United States; Veterans Affairs, Baltimore, Maryland, United States; University of Maryland School of Medicine, Baltimore, Maryland, United States

## Abstract

**Purpose:**

Functional limitations contribute to disability and fall risk in older adults. This analysis examined patterns of limitations over 12 months and if baseline function predicts falls in older Veterans participating in GeroFit, a supervised but unstructured VA exercise program.

**Methods:**

199 Veterans (71.9±6.7 years) completed functional assessments at baseline and 12 months (12M), including Gait Speed (GS), Short Physical Performance Battery (SPPB), Four Square Step Test (FSST), and 30-Second Chair Stand (30sCS). Functional limitations were defined by published cut-off scores. Paired t-tests and Wilcoxon signed-rank test assessed changes over time, chi-square tests examined co-occurrence, and logistic regression evaluated predictors of falls at 12M.

**Results:**

At baseline, 40% of participants had limitations in at least two measures, and at 12M, 30% remained below cut-off in multiple tests. Overall, Veterans improved significantly (p < 0.01) on all functional measures from baseline to 12M (SPPB: 10±2 to 11±2; GS: 1.10±0.26 m/s to 1.14±0.24 m/s; FSST: 13.12±6.90s to 11.63±6.54s; 30sCS 12±5 to 15±6 reps). Persisting below cut-offs was strongly predicted by baseline scores (OR = 2.58, 95% CI: 1.95–3.44, p < 0.001). Baseline 30sCS was the only significant predictor of falls at 12M (OR = 0.76, 95% CI: 0.56–0.98, p = 0.049), with each additional repetition in the 30sCS at baseline associated with a 24% lower odds of reporting a fall at 12 months.

**Conclusion:**

Lower extremity strength (30sCS) may be key for fall risk screening. Functional limitations frequently co-exist, and identification may guide interventions for older Veterans in exercise programs.

